# Bioactive lipids are altered in the kidney of chronic undernourished rats: is there any correlation with the progression of prevalent nephropathies?

**DOI:** 10.1186/s12944-017-0634-z

**Published:** 2017-12-16

**Authors:** Luzia S. Sampaio, Paulo A. da Silva, Valdilene S. Ribeiro, Carmem Castro-Chaves, Lucienne S. Lara, Adalberto Vieyra, M. Einicker-Lamas

**Affiliations:** 1 Instituto de Biofísica Carlos Chagas Filho – CCS, UFRJ, Rio de Janeiro, RJ Brazil; 2 Programa de Pós-Graduação em Biomedicina Translacional, Universidade do Grande Rio, Duque de Caxias, RJ Brazil; 3 Departamento de Fisiologia e Farmacologia, UFPE, Recife, PE Brazil; 4 Instituto de Ciências Biomédicas, UFRJ, Rio de Janeiro, RJ Brazil; 5 Centro Nacional de Biologia Estrutural e Bioimagem (CENABIO), UFRJ, Rio de Janeiro, Brazil; 6 Present Address: Laboratório de Biomembranas, Sala G1-037, Bloco G, Instituto de Biofísica Carlos Chagas Filho – CCS, UFRJ, Ilha do Fundão, Rio de Janeiro, RJ 21941-902 Brazil

**Keywords:** Bioactive lipids, Cholesterol, Kidney, Undernutrition, Lipid rafts

## Abstract

**Background:**

Undernutrition during childhood leads to chronic diseases in adult life including hypertension, diabetes and chronic kidney disease. Here we explore the hypothesis that physiological alterations in the bioactive lipids pattern within kidney tissue might be involved in the progression of chronic kidney disease.

**Methods:**

Membrane fractions from kidney homogenates of undernourished rats (RBD) were submitted to lipid extraction and analysis by thin layer chromatography and cholesterol determination.

**Results:**

Kidneys from RBD rats had 25% lower cholesterol content, which disturb membrane microdomains, affecting Ca^2+^ homeostasis and the enzymes responsible for important lipid mediators such as phosphatidylinositol-4 kinase, sphingosine kinase, diacylglicerol kinase and phospholipase A_2_. We observed a decrease in phosphatidylinositol(4)-phosphate (8.8 ± 0.9 vs. 3.6 ± 0.7 pmol.mg^−1^.mim^−1^), and an increase in phosphatidic acid (2.2 ± 0.8 vs. 3.8 ± 1.3 pmol.mg^−1^.mim^−1^), being these lipid mediators involved in the regulation of key renal functions. Ceramide levels are augmented in kidney tissue from RBD rats (18.7 ± 1.4 vs. 21.7 ± 1.5 fmol.mg^−1^.min^−1^) indicating an ongoing renal lesion.

**Conclusion:**

Results point to an imbalance in the bioactive lipid generation with further consequences to key events related to kidney function, thus contributing to the establishment of chronic kidney disease.

## Background

The underdeveloped or developing countries are those with the highest rates of undernutrition, which results from insufficient food intake, or even from an inappropriate diet [1]. Among the negative consequences from undernutrition, dyslipidaemia appears in a significant number of individuals, being the lipid disorders notably involved in different pathological conditions, here included coronary disorders, heart failure and other consequences [2, 3]. Therefore, different pharmacological and non-pharmacological interventions had already been proposed to healthy control of dyslipidaemia, being the latter represented by “nutraceuticals” and functional food for Review see [4]. In Brazil, the great diversification of dietary patterns is strongly related to differences in access to food, with different socio-economic profiles, culture and dietary habits of the population [5]. Chronic undernutrition is the most common form of nutritional deficiency, specially the observed during intrauterine, perinatal period and childhood, which are considered to be responsible for chronic illnesses in adulthood. Among them, cardiovascular alterations, arterial hypertension and nephropathies are frequently reported [1, 5–8].

In recent years, different studies from our group have demonstrated that undernutrition affected the kidney in different levels, compromising glomerular morphology and hemodynamic, changing Na^+^ renal transporter’s activities, being the amount of reabsorbed Na^+^ decisive in determining the volume and pressure of the circulating blood thus contributing to the establishment of hypertension [9–11].

The proximal tubule is a key nephron segment as it exhibits a broad range of hormones and autacoids receptors that participate in the modulation of the different active transporters (ATPases) including those responsible for Ca^2+^ and Na^+^ reabsorption [10–13]. In addition, there are other molecules that decisively participate in the modulation of these active transporters, such as the bioactive lipids [14–18]. In recent years, different glycerophospholipids and sphingolipids had emerged as important signaling molecules that play crucial roles in different kidney features. There are some reports that showed phosphatidylinositol-4 phosphate (PtdIns4P) as an important direct regulator of plasma membrane Ca^2+^-ATPase (PMCA) [14, 15]. Other studies had demonstrated that phosphatidic acid (PA) increases the ouabain-resistant Na^+^-ATPase activity upon angiotensin II treatment [18]. Concerning the sphingolipids, our group had recently demonstrated that ceramide (Cer) and ceramide-1-phosphate (C1P) are potent regulators of PMCA, Na^+^+K^+^-ATPase and Na^+^-ATPase in the basolateral membranes (BLM) from kidney proximal tubules [16, 17]. In addition, previous results from our group, had demonstrated that the PMCA was placed and active in caveolar microdomains within the BLM [19]. The assembly of these membrane microdomains is closely related to the cholesterol amount in the membrane rafts [20, 21]. Beyond its role in lipid rafts formation, cholesterol is also known to participate in the regulation of Na^+^+K^+^-ATPase [22]. Therefore, impairment in the cholesterol synthesis would disturb lipid rafts formation and consequently, inhibit the ion transporters cited above.

These observations led us to postulate that undernutrition would affect the bioactive lipids and cholesterol content in kidney cells, which in turn, would disrupt the physiological regulatory network responsible for ions and other solutes homeostasis. The aim of this study was to investigate a possible correlation between chronic malnutrition with changes in the pattern of formation of the different bioactive lipids and the cholesterol content in renal proximal tubules that would favors the functional changes already related to the Na^+^ active transporters.

## Methods

### Regional basic diet (RBD)

A complete and detailed comparison between RBD and the control balanced commercial rodent chow (Purina-Agribrands - Paulínia, SP, Brazil) was already published [23]. The components (in g%, wet weight) of control and RBD diets are, respectively: protein 23.0 and 7.9, carbohydrates 44.5 and 69.2, fat 2.5 and 0.8, fiber 7.2 and 8, mineral mix (except NaCl) 7.8 and 5.3, NaCl 0.16 and 0.33. Other nutritional information for RBD are according to Teodosio et al. (1990) [24]. The total kcal/100 g was 290 and 314 for control and BRD, respectively. The diet is composed of manioc flour (65 g/g%), brown beans (18 g/g%), sweet potato (13 g/g%) and jerked meat (4 g%). This isocaloric experimental diet was able to promote a pattern similar to that from the multifactorial malnutrition observed in humans [24].

### Animals

We used Wistar rats and management protocols with animals were approved by the Evaluation Committee on the Use of Animals in Research of the Instituto de Biofísica Carlos Chagas Filho (protocol N^o^ IBCCF 104), which is in accordance with the EU Directive 2010/63/EU. During pregnancy and postnatal periods, animals were kept in individual cages with free access to water and food. After the end of the lactation period (3 weeks) rat pups (all males) were divided into two experimental groups (8 to 10 animals per group), control and malnourished (RBD). Both groups had free access to food and water, and after the period of 13 weeks, where they show great reduction in body weight and blood pressure elevation [9, 25], the animals were euthanized.

#### Preparation of kidney proximal tubules cell membrane fraction

Preparation of a fraction enriched with basolateral membranes was carried out as previously described [23, 26]. Briefly, kidneys were collected and kept in cold isotonic.

buffer containing 250 mmol/l sucrose, 10 mmol/l HEPESTris (pH 7.4), 2 mmol/l EDTA, and 0.15 mg/ml trypsin inhibitor (Type II-S) supplemented with 1 mmol/l PMSF. Transverse thin slices (0.5 mm) of the external part of kidneys (cortex *corticis*) were removed using a Stadie-Riggs microtome (Thomas Scientific, Swedesboro, NJ, USA) and carefully dissected. The suspension was homogenized in the same cold.

buffer (4 ml/g) using a Teflon/glass homogenizer. The homogenate was centrifuged at 755×*g* (15 min) to sediment cell debris and nuclei; the resulting supernatant was centrifuged at 8,500×*g* (20 min) and at 35,000×*g* (45 min) at 4 °C. The final sediment was resuspended in 250 mM sucrose, aliquoted into tubes, and stored at −20 °C. After obtaining the material, total protein content was determined by the method described by Lowry et al. [27].

### Determination of Phosphatidylinositol-4 Kinase, Sphingosine Kinase and Diacylglycerol Kinase activities

The assay is based on the membrane lipid phosphorylation by^32^Pi (^32^Pi was from IPEN, São Paulo, Brazil) according to Einicker-Lamas et al. [28]. Briefly, an aliquot of the membrane fraction (0.1 mg total protein) was incubated as indicated with 0.1 mM sphingosine (Sph) (Sigma Chemical Co., Saint Louis, MO), then total lipid extraction was performed as described by Horwitz and Perlman [29]. PtdIns4P, sphingosine-1 phosphate (S1P) and PA were identified by thin layer chromatography (TLC) (chromatography silica gel 60 plates were from Merck, Darmstadt, Germany) followed by phosphorscreen analysis (Storm PhosphorImager 860 - Molecular Dynamics, Amersham Biosciences, Sunnyvale, CA). The bands corresponding to each lipid of interest were eluted, and counted using a liquid scintillation counter (Tri-Carb, Packard). Each experimental condition was assayed, at least, in three independent experiments, performed in triplicate as depicted in the respective legends to figures.

### Ceramide Kinase and Phospholipase A_2_ activities

To determine the endogenous levels of Cer and LPA, we performed the same assay based on membrane lipid phosphorylation described above, followed by lipid extraction and TLC analysis according to Bektas et al. [30]. C1P and lysophosphatidic acid (LPA) were identified using the phosphorscreen. The bands corresponding to these lipids were eluted, placed in vials and counted using a liquid scintillation counter (Tri-Carb, Packard). Result shows mean ± SEM of at least three different independent experiments performed in triplicate.

### Cholesterol determination

Briefly, an aliquot (0.1 mg total protein) from membrane fractions derived from kidney of the different experimental groups were used for total lipid extraction [28] and further cholesterol determination following the method described by Courchaine et al. [31]. Pure cholesterol (Sigma) was used as a standard. Each experimental set were performed independently (*n* ≥ 3, as shown in the respective legends to figures), in triplicate.

### SDS PAGE and immunodetection

Total protein aliquots (40 μg) from the different membrane fraction were separated in sodium dodecyl sulfate-polyacrylamide gels (10% and 12% for PMCA and Cav-1, respectively) and then transferred to nitrocellulose membranes at 350 mA. Immunodetection was performed using the following antibodies: PMCA H300 or CAV-1 H97 (Santa Cruz Biotechnology) at 1:500 dilution, 12 h. Then, the membranes were incubated with the secondary antibody (Peroxidase labeled ECL) 1:2000 dilution, for 2 h. The proteins of interest were detected using the ECL™ system and the Hyperfilm™ (Amershan, Buckinghamshire, UK).

### Statistical analysis

We used the software Graphpad Prism®5 and the results were expressed as mean ± SD (the number of experiments is depicted in the legend to each Figure). Values were submitted to Student’s t-test and, to compare more than two means the data were analyzed using one-way ANOVA with Tukey pos test. We established a *P* < 0.05 significance level.

## Results

### Cholesterol content is diminished in kidney cell membranes after chronic undernutrition

In a previous paper from our group, we showed that undernourished rats had no alteration in the levels of cholesterol either in serum or whole kidney homogenates, whereas it was significantly reduced in the plasma membranes from kidney proximal tubule cells [23]. This observation led us to verify if there could be any correlation between a reduced amount of cholesterol within the plasma membrane of kidney tubular cells and the normal ability of this plasma membrane to produce the bioactive lipids of interest here. Thus, we started to analyze the cholesterol content from our kidney membrane samples, not only due to its importance to cell membranes organization, dynamics and function, but also to its related action as a controller of the most important enzyme in kidney function – the Na^+^+K^+^-ATPase.

**Fig. 1:**
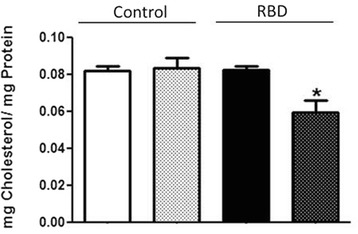
Reduced cholesterol amount in the kidney membrane fractions but not in whole cell homogenates from the RBD group. Graphical representation of the total cholesterol content in the whole cell homogenate (white bar), and membrane fraction (white crosshatched bar) from control group; and whole cell homogenate (black bar) and membrane fraction (black crosshatched bar) from RBD group. * < 0.05, where * is different from the control. Results are expressed as means ± SEM from different experiments done in triplicates (*n* = 10)

Figure 1 shows that the amount of cholesterol present in the RBD membrane fractions is significantly reduced when compared to the control (0.08 ± 0.01 vs. 0.05 ± 0.01 mg cholesterol/mg protein), whereas there was no alteration in the content of this lipid in whole cell homogenates (0.08 ± 0.005 vs. 0.08 ± 0.005 mg cholesterol/mg protein). This lower membrane cholesterol content could account for the inhibition of Na^+^+K^+^-ATPase as recently described by our group [9, 25] and also would imply in alterations in the plasma membrane moiety principally leading to a perturbation in the lipid rafts.

#### Lipid rafts disruption and altered targeting of plasma membrane Ca^2+^-ATPase (PMCA) to the kidney tubular cell membranes

**Fig. 2:**
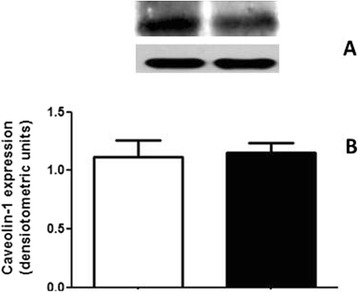
The caveolin-1 expression does not change in the RBD group. **a** Representative image from a western blotting for caveolin-1, together with the β-actin expression to ascertain protein load on the SDS-PAGE, as described in Methods. (**b**) Graphical representation of the caveolin-1 expression, normalized by the β-actin expression. Control, white bar and RBD, black bar. Results are expressed as means ± SEM from different experiments (*n* = 3)

**Fig. 3:**
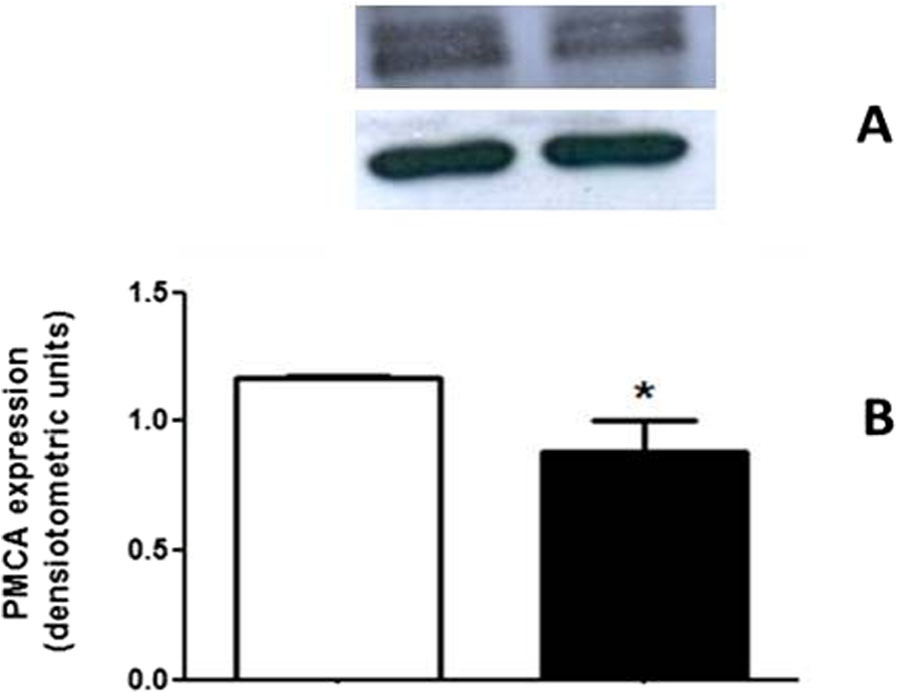
PMCA expression and targeted to the plasma membrane from kidney cells is significantly decreased in RBD group.** a** Representative image from a western blotting for PMCA, together with β-actin used for protein load control. **b** Graphical representation of the PMCA expression, normalized by the β-actin content. Control, white bar and RBD, black bar. Results are expressed as means ± SEM from different experiments (*n* = 3)

We attempted to access the impact of lower cholesterol amount in kidney cell membranes by measuring if there was any alteration in caveolae content. The caveolar microdomains are especially important for kidney cells as they harbor PMCA, which is responsible for the fine tuned regulation of intracellular Ca^2+^ and therefore, all the Ca^2+^-dependent signaling events that participates in the control of fluid transport across the renal membranes. When comparing the caveolae content in RBD with the control group (Fig. 2) we cannot observe any significant difference (1.1 ± 0.2 vs. 1.1 ± 0.1 densitometric units). However, it is clearly observed that there is a significant reduction in the PMCA content within the renal membranes in the undernourished group when compared to the control (1.1 ± 0.007 vs. 0.6 ± 0.2 densitometric units) (Fig. 3). The lowest content of cholesterol associated with a lowest availability of PMCA within the renal plasma membranes would account for an altered intracellular Ca^+^ concentration that would also alter the normal function of different enzymes involved in the generation of important bioactive molecules, here included the bioactive lipids of our interest. The next experiments were planned to access this issue.

### Bioactive lipid synthesis is altered upon chronic undernutrition

**Fig. 4:**
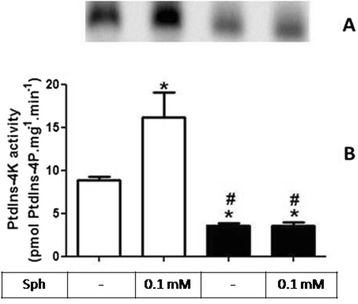
RBD diet intake causes a reduction in the PtdIns-4 K activity and loss of sph-induced modulation**. a** Representative image of a TLC for identification and quantification of PtdIns(4)P obtained as described in Methods. **b** Graphical representation of the PtdIns-4 K activity in kidney membrane fractions, determined by the measurements of PtdIns(4)P formation in the absence and presence of 0.1 mM Sph. White bars are control and black bars are RBD group. * and # < 0.05, where * is different the control and # different from the control and control + sph. Results are expressed as means ± SEM from different experiments (*n* = 4) done in triplicates

The result presented in Fig. 4 show that the phosphatidylinositol-4 kinase (PtdIns-4 K) activity, which result in the formation of PtdIns(4)P, is significantly reduced in the RBD group (8.8 ± 0.9 vs 3.6 ± 0.7 pmol PtdIns(4)P.mg^−1^.mim^−1^). We also demonstrated that the stimulation of PtdIns-4 K activity by sphingosine (Sph) previously described by our group [28] and also here (8.8 ± 0.9 vs. 16.1 ± 4.0 pmol PtdIns(4)P.mg^−1^.mim^−1^) was completely abolished in the RBD group (3.6 ± 0.7 vs. 3.5 ± 1.2 pmol PtdIns(4)P.mg^−1^.mim^−1^).

**Fig. 5:**
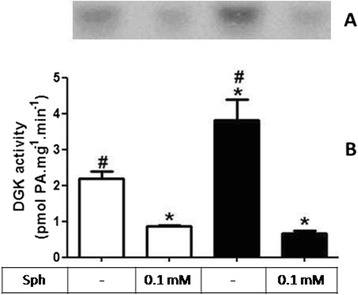
RBD causes an increase in the DGK activity but do not alters the sph-induced modulation in this lipid kinase**. a** Representative image of a TLC for identification and quantification of PA formed, as described in Methods. **b** Graphical representation of the DGK activity in membrane fractions, calculated from the formation of PA in the absence and presence of 0.1 mM sph. White bars are control and black bars are RBD group. * and # < 0.05, where * is different the control and # different from the control and control + sph. Results are expressed as means ± SEM from different experiments (n = 4) done in triplicate

Another key enzyme in the generation of a bioactive lipid studied was the diacylglycerol kinase (DGK), which is responsible for controlling the balance between PA and diacylglycerol (DAG), being both bioactive lipids with important signaling functions. The result presented in Fig. 5 shows that DGK activity is significantly increased in the RBD group, leading to a higher formation of PA (2.2 ± 0.4 vs. 3.8 ± 1.3 pmol PA.mg^−1^.mim^−1^). Different from the observed for PtdIns-4 K, the inhibition of DGK by Sph [28] was preserved in both groups tested (2.2 ± 0.4 vs. 0.8 ± 0.05 X 3.8 ± 1.3 vs.0.6 ± 0.1 pmol PA.mg^−1^.mim^−1^).

**Fig. 6:**
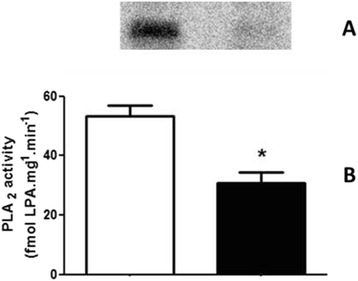
Undernutrition leads to a significant decrease in PLA_2_ activity**. a** Representative image of a TLC for identification and quantification of LPA formation, obtained as described in Methods. **b** Graphical representation of the PLA_2_ activity in the membrane fractions, calculated from the conversion of PA to LPA. White bar is control and black bar is RBD group. * < 0.05, where * is different from the control. Results are expressed as means ± SEM from different experiments (*n* = 6) done in triplicate

The augmented content of PA available could contribute to an increase in the generation of LPA as a result of phospholipase A_2_ (PLA_2_) activity, which would also alters the physiological modulation of different kidney functions including the absorption processes mainly through different ion transporters. To investigate this point, we performed an experimental set to determine the PLA_2_ activity through the quantification of LPA formed. Figure 6 clearly shows that membrane fractions from the RBD group had a significant reduction in LPA formation (53.3 ± 9.3 vs. 30.8 ± 9.4 fmol LPA.mg^−1^.mim^−1^).

**Fig. 7:**
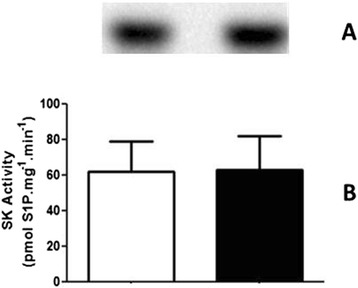
The SK activity does not change in the RBD group. **a** Representative image of a TLC for identification and quantification of the S1P formation, obtained as described in Methods. **b** Graphical representation of the SK activity in kidney membrane fractions, calculated from the S1P formation.White bar is the control and black bar the RBD group. Results are expressed as means ± SEM from different experiments (n = 4) done in triplicates

**Fig. 8:**
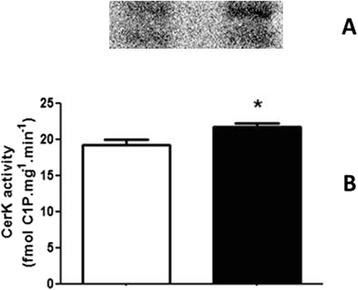
Increased CerK activity in the RBD group. **a** Representative image of a TLC for identification and quantification of C1P formation obtained as described in Methods. **b** Graphical representation of the CerK activity in membrane fractions, calculated from the formation of C1P. White bar is the control and black bar the RBD group. * < 0.05, where * is different from the control. Results are expressed as means ± SEM from different experiments done in triplicates (n = 6)

Bioactive sphingolipids were also analyzed here, especially Sph, Cer and their phosphorylated derivatives S1P and C1P, respectively, due to their involvement in the regulation of cell signaling pathways that are known to modulate ion transporters in kidney proximal tubule cells. Figure 7 shows that S1P was not altered in the RBD group when compared to the control (61.6 ± 37.9 vs. 62.9 ± 42.2 pmol S1P.mg^−1^.mim^−1^), while there was a slightly but significant increase in Cer availability (Fig. 8), here accessed by the formation of C1P (18.7 ± 1.4 vs. 21.7 ± 1.5 fmol C1P.mg^−1^.mim^−1^).

These results shows for the very first time that the RBD diet leads to a very important lipid remodeling in kidney cells, and this altered pattern of bioactive and structural lipids may be part of the overall physiological changes that occurs in chronic undernourished animals.

## Discussion

The harmful effects of chronic undernutrition in different tissues and organs are clearly associated to several chronic diseases observed in human population principally in adulthood. Meanwhile, the cellular basis for these physiological alterations and in some extent, cellular events that occur during or leading to metabolic programming, are still not fully understood. One of the principal mechanisms affected by undernutrition is the ion transport control, which is directly associated to some important pathologies such as hypertension and other cardiac alterations, diabetes and renal failure [1, 5–8,10]. In recent years, our group gave some contributions to the field, which aimed to point the regulatory pathways involved in the patophysiological alterations observed in undernourished rats. Silva et al. (2014) had demonstrated an imbalance in the PKC and PKA-mediated pathways, with participation of angiotensin receptors and activation of the MAPK/ERK1/2 pathway [9]. These molecular and cellular alterations lead to cardiac electric remodeling and contribute to the onset of hypertension in adulthood. We had also demonstrated that the undernourished group presented a 34% reduction in body weight at birth, being this difference superior to 70% after 60 days [25]. Other contributions from our group had shown that there is a significant infiltrate of macrophages which is accompanied by a higher deposition of collagen with further increase in renal fibrosis in the undernourished group [32, 33].

In parallel to some of the above referred studies, we started to investigate the role of the bioactive lipids in the onset of the metabolic programming in kidney from undernourished rats. We based our research proposal on the fact that among different cell signaling pathways and second messengers involved in the regulation of most of the metabolic cell machinery, bioactive lipids generation and a possible disturb in their proper synthesis and action, would alter other different physiological fates leading to the harmful effects observed in some prevalent nephropathies. The overall alteration in the bioactive lipids pattern in the RBD group would account for the impairment or altered activity of Na^+^ transporters in the renal epithelium such as, the Na^+^+K^+^-ATPase and the Na^+^-ATPase. Our first evidence of the involvement of lipids in the renal tissue imprint caused by RBD was the significant reduction in cholesterol found in plasma membrane from kidney tubular cells [24]. We considered that this observation of a significant lower content of cholesterol in the membranes from RBD kidney cells (see Fig. 1) would be sufficient to disrupt or to importantly alter the dynamics of assembly of membrane microdomains – the lipid rafts [19, 34]. It is well known that these rafts are key membrane regions for different cell signaling pathways and also to different ion transporters, principally the PMCA which was related to be exclusively placed and active within the caveolar microdomains in renal cortical membranes [19]. Thus, although the amount of caveolae in the renal membranes is not altered in the RBD group (Fig. 2), there is a significant lower content of PMCA in these membranes, which may account for an abnormal increase in the intracellular Ca^2+^ content, leading to the deregulation of several Ca^2+^-dependent events. We can assume that this deficiency in Ca^2+^ homeostasis would contribute to the altered production of important bioactive lipids. For example, PtdIns-4 K is strongly inhibited in high Ca^2+^ condition resulting in a decrease in PtdIns(4)P formation, which is important to regulate the activity of PMCA and other P-type ATPases [14, 35, 36]. In addition, the reduction in PtdIns(4)P synthesis would interfere with the normal phosphoinositide cycle probably repercuting in a myriad of cell signaling pathways triggered by different hormones and autacoids that activates phospholipase C and further, protein kinase C (PKC). It is demonstrated that Na^+^+K^+^-ATPase and Na^+^-ATPase are differentially affected in undernourished rats: while there is an inhibition in the Na^+^+K^+^-ATPase activity, Na^+^-ATPase activity is significant augmented [9, 25]. These effects verified on the active Na^+^ transporters may be explained at least by two facts: (i) reduction in cholesterol content, which is important for Na^+^+K^+^-ATPase activity [22]; and (ii) failure in PKC driven regulation of key cellular processes as a result of reduced phosphoinositide turnover. Another point to be discussed is the disruption of the crosstalk between glycero- and sphingolipids already described in these renal membranes [28] that should be a crucial event for the cell membrane integrity and stability. Here we demonstrated that PtdIns(4)P is significantly reduced, accompanied to a shunt in the activation of S1P synthesis that is normally observed (Fig. 4). We can suggest that the altered membrane dynamics, with disruption of part of the lipid rafts as a result of decreased cholesterol content, would be the reason of the uncoupled bioactive lipids machinery. These alterations would imply in the progression of cell damage. To support this view, Fig. 8 shows the detection of higher amounts of ceramide (Cer) in the RBD group. Cer is referred to cell death and more recently it is also implicated in the activation of important cell signaling pathways, including the activation of protein kinase A (PKA) and also PKCζ [16, 17], being the latter associated to different harmful effects in different pathologies including cancer, cardiovascular diseases and nephropathies [37, 38].

In resume, we can strongly suggest that the pivotal role of cholesterol in the assembly of membrane microdomains is a primary harmful effect observed in the RBD group, which will result in the altered pattern of bioactive lipids that culminates with impaired maintenance of ion homeostasis and cell physiology. Therefore we can assume that the chronic diseases present in adulthood would be caused by a disruption in the regulatory network involving glycero- and sphingolipids in kidney cells.

## Conclusions

Multifactorial malnutrition causes significant changes in the function of renal epithelium as a consequence of altered production and distribution of the mainly bioactive lipids. It is noteworthy that the change in the cholesterol content at the cell membrane can disrupt other structures that are normally well assembled to facilitate the operation of the basic machinery on the renal tubular epithelium, here included the cellular signaling pathways and the triggered ion transporters. All data discussed here, with others from the literature, particularly on the renal function loss and changes in ion transport across the proximal tubular epithelium, show that the bioactive lipids are closely related to the overall tissue damage caused by chronic undernutrition.

## Abbreviations

BLM: Basolateral membranes
C1P: Ceramide-1-phosphate
Cer: Ceramide
CerK: Ceramide kinase
DAG: Diacylglycerol
DGK: Diacylglycerol kinase
LPA: Lysophosphatidic acid
PA: Phosphatidic acid
PKA: Protein kinase A
PKC: Protein kinase C
PLA_2_: Phospholipase A_2_
PMCA: Plasma membrane Ca^2+^-ATPase
PtdIns-4 K: Phosphatidylinositol-4 kinase
PtdIns4P: Phosphatidylinositol-4 phosphate
RBD: Regional Basic Diet
S1P: Sphingosine-1 phosphate
SK: Sphingosine kinase
Sph: Sphingosine
TLC: Thin layer chromatography
